# Liver transplantation combined with tyrosine kinase inhibitors for the treatment of hepatic metastatic giant gastrointestinal stromal tumors: A case report and literature review

**DOI:** 10.3389/fgstr.2022.884507

**Published:** 2022-08-31

**Authors:** Jun Lin Qian, Ze Min Hu, Kun He, Yong Zhu He

**Affiliations:** ^1^ Department of Hepatobiliary Surgery, Zhongshan Hospital Affiliated to Sun Yat-sen University, Zhongshan, China; ^2^ Graduate School of Hepatobiliary Surgery, Guangdong Medical University, Zhanjiang, China; ^3^ Department of Hepatobiliary Surgery, The First Affiliated Hospital of Nanchang University, Nanchang, China

**Keywords:** gastrointestinal stromal tumor (GIST), liver metastases, liver transplantation, tyrosine kinase inhibitor (TKI), imatinib

## Abstract

**Background:**

Surgical resection combined with oral tyrosine kinase inhibitors(TKI) is the most effective treatment for gastrointestinal stromal tumor(GIST) liver metastases. Liver transplantation (LT) is the last resort for the treatment of terminal liver malignancy. Whether it can be a potential treatment option for liver metastases from unresectable GIST is worth exploring.

**Case presentation:**

We report a 38-year-old woman who underwent jejunal stromal tumor resection and TKI(imatinib) therapy 15 years ago for jejunal stromal tumor liver metastases. During the period from 2015 to 2018, the liver metastases continued to grow after the patient stopped taking imatinib voluntarily, and the patient subsequently underwent multiple interventional surgeries and drug treatments, which were still poorly curative. The tumor was deemed unresectable because it filled the entire liver, and the patient subsequently underwent LT and was treated with imatinib post-operatively, which resulted in no recurrence of the tumor within 18 months of follow-up.

**Literature review:**

There are few reports in the literature on LT for the treatment of liver metastases from GIST. A systematic review and summary of the current literature by literature search revealed that LT as a last resort for metastatic GIST of the liver remains a major challenge.

**Conclusions:**

LT combined with TKI-targeted therapy is a potential therapy worth exploring for patients with unresectable metastatic GIST.

## Introduction

The liver is the most common metastatic site of gastrointestinal stromal tumors (GIST), and about 15%-20% of GIST patients develop synchronous liver metastases (1). Therefore, the degree of control of liver metastases is one of the main factors determining the long-term survival of such patients (2). At present, some studies believe that the most effective treatment for liver metastatic GIST is surgical resection combined with oral tyrosine kinase inhibitors(TKI), which can significantly prolong the survival time of patients (3, 4). For patients with TKI-resistant and unresectable liver metastatic GIST, there has been a lack of effective treatment measures. Liver transplantation (LT) is the last resort for the treatment of end-stage liver malignancies, and its combination with TKI-targeted therapy may prolong the survival time of such patients (5–7). In this report, we present a patient with unresectable giant hepatic metastatic GIST who received LT combined with TKI therapy, and discuss the value of this treatment option for this group of patients in combination with the literature.

## Case report

**Figure 1:**
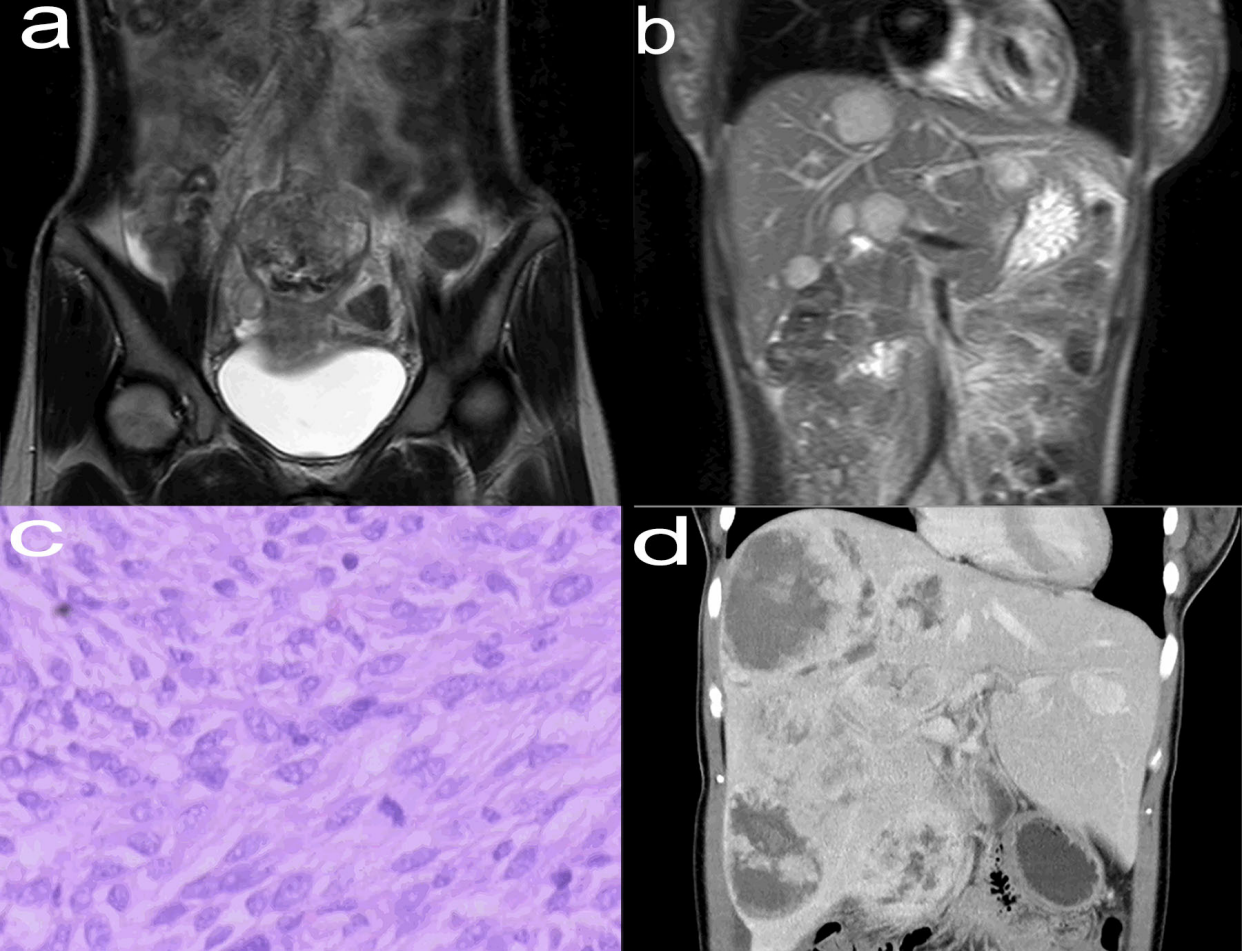
Enhanced MRI (May 2005) of the abdomen (coronal view) showed a thickened jejunum intestinal tube wall above the uterus, cauliflower-like mass in the cavity, unclear boundary between the lesion and the surrounding tissue structure, and the range was about 6.5 cm × 6.2 cm × 6.4 cm **(A)**. Enhanced MRI (May 2005) of the abdomen (coronal view) showed that the liver was filled with nodules of different sizes and inconsistent signals, with clear margins and a maximum range of about 3.4 cm×3.0 cm × 3.0 cm **(B)**. Typical pathological images of jejunal stromal tumor (May 2005, hematoxylin and eosin stain, ×200) **(C)**. Enhanced CT (August 2018) of the abdomen (coronal view) revealed multiple liver metastases, some solid and some cystic, with a maximum diameter of about 11.5 cm **(D)**. MRI, Magnetic Resonance Imaging; CT, Computed Tomography.

A 38-year-old woman was admitted to our hospital 15 years ago with progressive abdominal pain. Enhanced Magnetic Resonance Imaging (MRI) showed a cauliflower-like mass in the jejunum above the uterus (**Figure 1A**) and nodules of different sizes in the liver (**Figure 1B**). The patient subsequently underwent palliative jejunal tumor resection. Pathology revealed atypical spindle cells and jejunal stromal tumor with multiple intrahepatic metastases was diagnosed (**Figure 1C**). Gene detection showed that exon 11 of c-kit gene was mutated. After operation, the patient took imatinib mesylate 0.4g/d and was followed up regularly.

**Figure 2:**
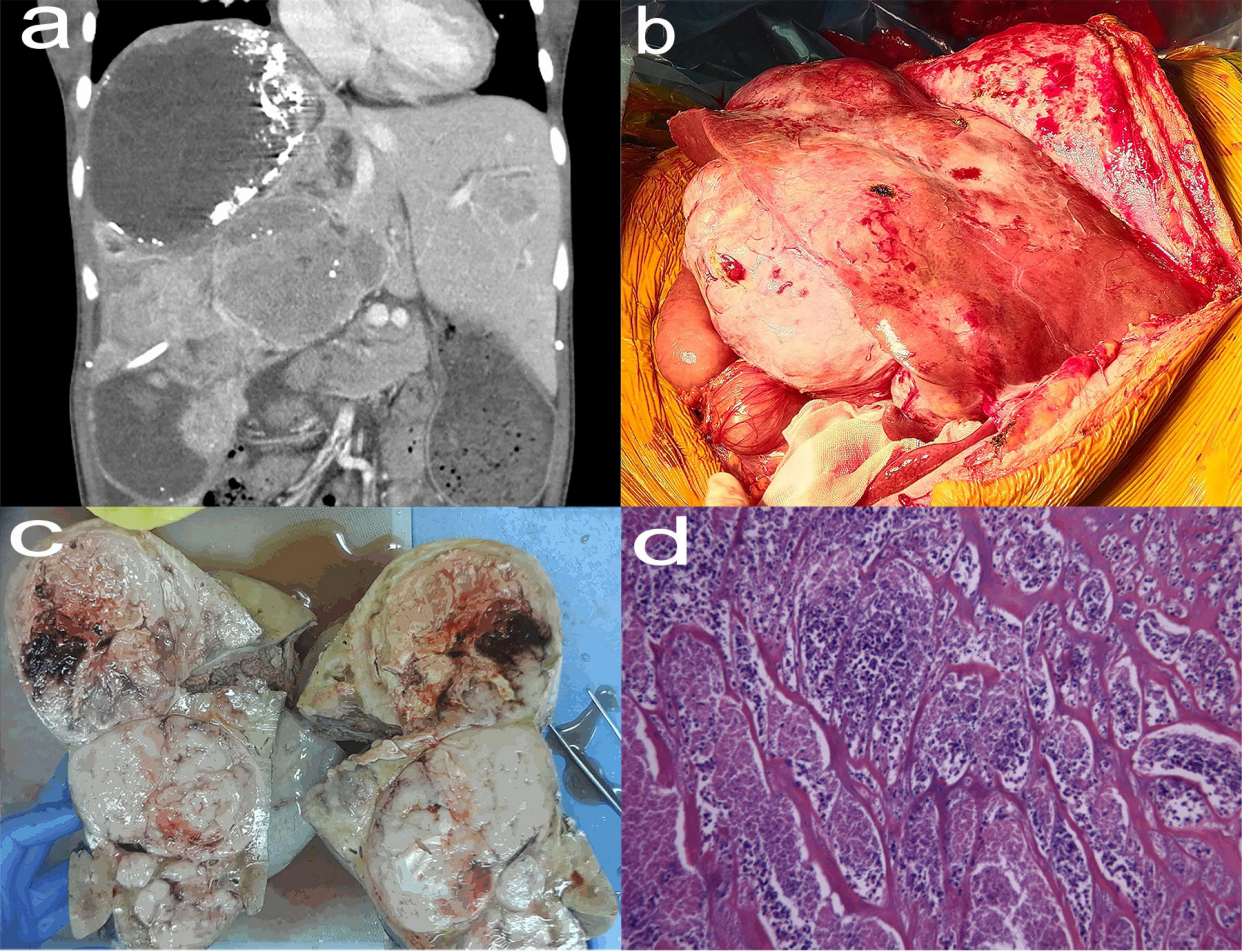
Enhanced CT (January 2021) of the abdomen (coronal view) showed that multiple tumors were in a fusion trend, basically occupying the entire right lobe of the liver, with a maximum diameter of about 20.0 cm **(A)**. Huge liver tumors seen in liver transplants **(B)**. Postoperative specimens showed multiple tumors that did not break through the liver capsule with a range of about 26 cm × 20 cm × 9 cm **(C)**. The tumor cells were arranged in bundles and braided shape, and the cells were long and fusiform, with necrosis, hemorrhage and 13 mitoses/50 high-power field, which was consistent with the typical pathological manifestations of jejunal stromal tumor (hematoxylin and eosin stain, ×200) **(D)**.

The patient self-discontinued imatinib mesylate tablets between June 2015 and August 2018, which resulted in recurrent abdominal pain and hospitalization. Enhanced Computed Tomography (CT) of the patient showed that the multiple tumors in the liver were significantly larger than before (**Figure 1D**). The patient continued to take imatinib mesylate tablets and underwent multiple interventional procedures, which did not lead to remission. Finally, the patient’s drugs were replaced with sunitinib malate and regorafenib in turn, but her condition still did not improve. The patient underwent orthotopic LT in January 2021, based on considerations of medication, interventional therapy, and enhanced CT (**Figure 2A**) showing progressively larger liver metastases that could not be completely resected. During liver transplantation, large intrahepatic metastases were observed without gastrointestinal recurrence or extrahepatic metastases (**Figure 2B**). Multiple large liver tumors were found in the postoperative specimen (**Figure 2C**), and the pathological diagnosis was gastrointestinal stromal tumor with liver metastasis (**Figure 2D**). Finally, genetic testing of the patient still suggested a mutation in exon 11 of the c-kit gene. After the operation, we gave the patient the immunosuppressant combined with the original regimen of imatinib mesylate. In the absence of other interventions, the patient has not had tumor recurrence at the current follow-up of nearly 18 months.

## Literature review

There are few reports in the literature on LT for the treatment of liver metastatic GIST. We searched the literature to analyze and summarize the clinical data of 8 patients who have reported LT for the treatment of unresectable liver metastatic GIST (**Table 1**). Husted et al (10). Reported a study of 13 patients with metastatic sarcoma, 8 of which were high-risk liver metastatic GIST, and found that these patients had early tumor recurrence and short median survival(only 10.8 months) after LT. Cameron et al (9). reported 2 patients with metastatic GIST who underwent LT, and their postoperative disease-free survival rates were 48 months and 69 months, respectively. Bompas et al (8). also reported a case of 24-month disease-free survival after LT. More encouragingly, Frilling et al (6). Reported that a patient with extragastrointestinal stromal tumor of liver metastases (17 years after primary tumor resection) after LT, although there was a 2 cm stable perirectal metastasis tumor, but remained asymptomatic for 10 years.

**Table 1:** Results of literature search on liver transplantation for unresectable GIST.

Study author	gender	Age of onset (years)	Time of onset/time of liver lesions	Source	Preoperative targeted drug selection	Year of liver transplantation	Postoperative targeted drug selection	Postoperative pathological immunohistochemistry	Postoperative disease-free survival/postoperative survival time
Bompas et al. (8)	–	–	1995/1995	Small intestine	NO	1995	Imatinib	C-kit 11 +	2 years/-
Frilling et al. (6)	Female	56	1992/1995	Rectum	NO	1999	Imatinib	CD34+, C-kit 11 +	2 years/10 years
Cameron et al. (9)	Male	39	1996/2000	**Stomach**	NO	2000	–	–	48 months/-
	Female	29	1989/1997	**Stomach**	NO	1999	–	–	69 months/-
Serralta et al. (5)	Female	41	1991/1995	Duodenum	NO	1995	Imatinib	C-kit 11 +	60 months/92 months
	Male	36	1999/1999	**Stomach**	NO	1999	Imatinib	C-kit 11 +	34 months/48 months
	Male	45	1998/1999	Duodenum	NO	1999	Imatinib	C-kit 11 +	21 months/46 months
Iesari et al. (7)	Female	16	2006/2007	Stomach	Imatinib, sunitinib, sorafenib	2015	–	C-kit 11 +, Dog1+, cd34-	4 years/-

In the past, the median survival time of patients with unresectable liver metastatic GIST was only 10-20 months, and it was rare to survive more than 5 years (11). DeMatteo et al (12). Reported 56 patients with liver metastases from sarcoma, 61% of which were GIST, and the 5-year survival rate after complete resection in most cases was 30%, compared with only 4% in unresection. Current international guidelines for diagnosis and treatment recommend that patients with GIST requiring combined organ resection receive targeted therapy first to reduce the risk of surgery and improve prognosis (13). For patients with unresectable metastatic GIST, imatinib is also a first-line treatment, and surgery can still be performed after tumor downstaging (14). LT is the only effective treatment option for patients with advanced hepatic malignancies, but its role in the treatment of metastatic hepatic malignancies remains controversial (10). Currently, for patients with TKI-resistant, unresectable hepatic metastatic GIST, LT may be the only treatment that can improve the prognosis after poor palliative treatment such as radiotherapy, chemotherapy, ablation, and hepatic arterial chemoembolization (5, 10, 15).

## Discussion

With the advent of the TKI imatinib, the concept of surgical treatment and comprehensive treatment of GIST has been completely changed (16). For recurrent and/or metastatic GIST, imatinib is recommended as first-line treatment, with a 2-year progression-free survival rate of 77%, but tumor shrinkage rate of only 4.5%, while with imatinib treatment with the prolongation of time, the proportion of drug resistance is also increasing, and no more than 5% of patients achieve complete remission (5–7, 16–18). It has been reported that the overall incidence of imatinib resistance is about 56%, and secondary resistance generally occurs 2-3 years after imatinib treatment (19). Because targeted therapy may eventually develop resistance, drug therapy alone cannot provide long-term benefits for patients with advanced GIST. Therefore, during the period of targeted therapy control, timely surgical treatment will help improve the survival and prognosis of patients. A number of studies have shown that for patients with resectable metastatic GIST, targeted therapy combined with surgical resection can significantly improve the overall survival rate and prolong disease-free survival compared with surgery or targeted therapy alone (20–22).

One patient reported in this study had liver metastatic GIST originating in the jejunum, which is unique not only in its clinical rarity, but also in the remission interval of more than 10 years. Unresectable liver metastases occurred when the patient was first discovered. Due to factors such as the medical technology at the time and the patient’s economic situation, the patient’s liver metastases could not be treated in time, and only jejunectomy combined with postoperative imatinib was performed on the patient. At the same time, the patient stopped taking imatinib voluntarily from 2015 to 2018, which led to the continuous growth of liver metastases. Even if imatinib was continued again, the tumor did not shrink. Studies have shown that for advanced GIST patients with imatinib treatment failure, imatinib can be replaced with sunitinib, and when both drugs fail, replacing regogfinil can also significantly improve the progression-free survival of these patients (23, 24). However, after the patient developed imatinib resistance, successive substitutions of sunitinib and regorafenib failed to improve the patient’s liver metastases enlargement. Therefore, our decision to perform LT in this patient after a multidisciplinary team discussion was based on an exhaustive preoperative evaluation and strict selection criteria: ①Unresectable liver metastases assessed by imaging; ②Unresponsive or intolerable to targeted therapy and other palliative treatments; ③No recurrence of primary tumor and no manifestations of extrahepatic metastasis; ④The interval between resection of primary tumor and remission of liver metastasis is longer (more than 2 years); ⑤The overall health status is good after supportive treatment and can tolerate liver transplantation; ⑥The compliance with long-term treatment with immunosuppressive agents and molecular targeted drugs is good. Of all the above criteria, many of the selection criteria were also recommended by Frilling et al (6).

The high recurrence rate after primary and metastatic GIST means that GIST is a systemic disease and its perioperative adjuvant therapy is very important. The timing and duration of initiation of adjuvant TKI therapy after LT is currently difficult to determine. In general, TKI therapy can be started as soon as high-risk patients recover from surgery and should be continued for 2 years (25). Gene mutation detection can make the treatment of such patients more individualized, and mutations mostly occur in KIT exon 11 (70%), KIT exon 9 (10%) and PDGFRA exon 18 (5%) (24, 25). Imatinib mesylate has a significant effect on c-kit exon 11 mutation, but not on c-kit exon 9 mutation and c-kit/PDGFRA wild type. In particular, the 842V mutation of PDGFRA may be resistant to primary imatinib mesylate (25, 26). In addition, sunitinib and regafinil are more beneficial for patients with exon 9 mutation and exon 17 mutation of N822K gene (23–26). Studies have shown that patients with GIST with c-kit 11 exon mutation should be the first choice for imatinib 400 mg/d as a preoperative, postoperative adjuvant therapy, or as a salvage drug for patients who cannot be resected (26). On the other hand, a D842V mutation in PDGFRA exon 18 leads to resistance to imatinib and sunitinib in patients. Fortunately, Avapritinib has proven to be the standard therapy for these patients (27). The genetic test of the patients in this study showed c-kit 11 exon mutation, and the patient was given oral imatinib after transplantation, which was well tolerated and had no obvious side effects.

The patient in this case was followed up for 18 months after LT, and no tumor recurrence has occurred so far. combined with the reported literatures, our preliminary experience suggests that LT combined with TKI-targeted therapy is a potential treatment worth exploring for unresectable metastatic GIST patients. However, the case in this study is only one case and the follow-up time is not long, and a multi-center large-sample study is needed to further evaluate the value of LT for the treatment of such patients.

## Data availability statement

The original contributions presented in the study are included in the article/supplementary material. Further inquiries can be directed to the corresponding authors.

## Ethics statement

Written informed consent was obtained from the participant for the publication of this case report.

## Author contributions

JQ and ZH participated in the clinical data and image collection and/or wrote the manuscript. KH is the main person in charge of the liver transplant operation. YH is in charge of data and image revision, text revision and content discussion of the entire manuscript. All authors contributed to the article and approved the submitted version.

## Funding

This work was supported by funding from Zhongshan Science and Technology Plan Project of Guangdong Province (Project Number: 2021B1040).

## Conflict of interest

The authors declare that the research was conducted in the absence of any commercial or financial relationships that could be construed as a potential conflict of interest.

## Publisher’s note

All claims expressed in this article are solely those of the authors and do not necessarily represent those of their affiliated organizations, or those of the publisher, the editors and the reviewers. Any product that may be evaluated in this article, or claim that may be made by its manufacturer, is not guaranteed or endorsed by the publisher.

## References

[1] Lillemoe HA Brudvik KW Vauthey JN. Treatment options for metastatic gastrointestinal stromal tumors to the liver: A review. Semin liver Dis (2019) 39(3):395–402. doi: 10.1055/s-0039-1685517 31100757

[2] Shi YN Li Y Wang LP Wang ZH Liang XB Liang H. Gastrointestinal stromal tumor (GIST) with liver metastases: An 18-year experience from the GIST cooperation group in north China. Medicine (2017) 96(46):e8240. doi: 10.1097/MD.0000000000008240 29145240 PMC5704785

[3] Ye YJ Gao ZD Poston GJ Wang S. Diagnosis and multi-disciplinary management of hepatic metastases from gastrointestinal stromal tumour (GIST). Eur J Surg Oncol (2009) 35(8):787–92. doi: 10.1016/j.ejso.2009.01.003 19185444

[4] Nanji S Cleary S Ryan P Guindi M Selvarajah S Al-Ali H. Up-front hepatic resection for metastatic colorectal cancer results in favorable long-term survival. Ann Surg Oncol (2013) 20(1):295–304. doi: 10.1245/s10434-012-2424-1 23054102

[5] Serralta AS Sanjuan FR Moya AH Orbis FC López-Andújar R Pareja EI. Combined liver transplantation plus imatinib for unresectable metastases of gastrointestinal stromal tumours. Eur J Gastroenterol Hepatol (2004) 16(11):1237–9. doi: 10.1097/00042737-200411000-00025 15489588

[6] Frilling A Malago M Testa G Schleyer E Grabellus F Kronenberger R. Liver transplantation for metastasized extragastrointestinal stromal tumor: a case report and an overview of literature. Transplant Proc (2010) 42(9):3843–8. doi: 10.1016/j.transproceed.2010.06.016 21094867

[7] Iesari S Mocchegiani F Nicolini D Benedetti Cacciaguerra A Coletta M Montalti R. Liver transplantation for metastatic wild-type gastrointestinal stromal tumor in the era of molecular targeted therapies: Report of a first case. Am J Transplant (2019) 19(10):2939–43. doi: 10.1111/ajt.15377 30943317

[8] Bompas E Boillot O Bringuier PP Dumortier J Blay JY. Imatinib in patients with metastatic gastrointestinal stromal tumours relapsing after hepatic transplantation. Eur J Cancer (Oxford England:1990) (2004) 40(9):1456–7. doi: 10.1016/j.ejca.2004.03.002 15177508

[9] Cameron S Ramadori G Füzesi L Sattler B Gunawan B Müller D. Successful liver transplantation in two cases of metastatic gastrointestinal stromal tumors. Transplantation (2005) 80(2):283–4. doi: 10.1097/01.TP.0000164141.34293.6B 16041278

[10] Husted TL Neff G Thomas MJ Gross TG Woodle ES Buell JF. Liver transplantation for primary or metastatic sarcoma to the liver. Am J Transplant (2006) 6(2):392–7. doi: 10.1111/j.1600-6143.2005.01179.x 16426326

[11] Kee D Zalcberg JR. Current and emerging strategies for the management of imatinib-refractory advanced gastrointestinal stromal tumors. Ther Adv Med Oncol (2012) 4(5):255–70. doi: 10.1177/1758834012450935 PMC3424497 22942908

[12] DeMatteo RP Shah A Fong Y Jarnagin WR Blumgart LH Brennan MF. Results of hepatic resection for sarcoma metastatic to liver. Ann Surg (2001) 234(4):discussion 547–548. doi: 10.1097/00000658-200110000-00013 PMC1422077 11573047

[13] Wardelmann E Thomas N Merkelbach-Bruse S Pauls K Speidel N Büttner R. Acquired resistance to imatinib in gastrointestinal stromal tumours caused by multiple KIT mutations. Lancet Oncol (2005) 6(4):249–51. doi: 10.1016/S1470-2045(05)70097-8 15811621

[14] Qiu HB Zhou ZG Feng XY Liu XC Guo J Ma MZ. Advanced gastrointestinal stromal tumor patients benefit from palliative surgery after tyrosine kinase inhibitors therapy. Medicine (2018) 97(2):e9097. doi: 10.1097/MD.0000000000009097 29480823 PMC5943843

[15] Machairas N Prodromidou A Molmenti E Kostakis ID Sotiropoulos GC. Management of liver metastases from gastrointestinal stromal tumors: where do we stand? J gastrointest Oncol (2017) 8(6):1100–8. doi: 10.21037/jgo.2017.08.08 PMC5750192 29299371

[16] Poveda A García Del Muro X López-Guerrero JA Cubedo R Martínez V Romero I. GEIS guidelines for gastrointestinal sarcomas (GIST). Cancer Treat Rev (2017) 55:107–19. doi: 10.1016/j.ctrv.2016.11.011 28351781

[17] Casali PG Le Cesne A Velasco AP Kotasek D Rutkowski P Hohenberger P. Final analysis of the randomized trial on imatinib as an adjuvant in localized gastrointestinal stromal tumors (GIST) from the EORTC soft tissue and bone sarcoma group (STBSG), the Australasian gastro-intestinal trials group (AGITG), UNICANCER, French sarcoma group (FSG), Italian sarcoma group (ISG), and Spanish group for research on sarcomas (GEIS). Ann Oncol (2021) 32(4):533–41. doi: 10.1016/j.annonc.2021.01.004 33482247

[18] Cavnar MJ Seier K Curtin C Balachandran VP Coit DG Yoon SS. Outcome of 1000 patients with gastrointestinal stromal tumor (GIST) treated by surgery in the pre- and post-imatinib eras. Ann Surg (2021) 273(1):128–38. doi: 10.1097/SLA.0000000000003277 PMC6774913 30946076

[19] Kelly CM Gutierrez Sainz L Chi P. The management of metastatic GIST: current standard and investigational therapeutics. J Hematol Oncol (2021) 14(1):2. doi: 10.1186/s13045-020-01026-6 33402214 PMC7786896

[20] Charo LM Burgoyne AM Fanta PT Patel H Chmielecki J Sicklick JK. A novel PRKAR1B-BRAF fusion in gastrointestinal stromal tumor guides adjuvant treatment decision-making during pregnancy. J Natl Compr Cancer Network (2018) 16(3):238–42. doi: 10.6004/jnccn.2017.7039 PMC6053908 29523662

[21] Raut CP Espat NJ Maki RG Araujo DM Trent J Williams TF. Efficacy and tolerability of 5-year adjuvant imatinib treatment for patients with resected intermediate- or high-risk primary gastrointestinal stromal tumor: The PERSIST-5 clinical trial. JAMA Oncol (2018) 4(12):e184060. doi: 10.1001/jamaoncol.2018.4060 30383140 PMC6440723

[22] Ling JY Ding MM Yang ZF Zhao YD Xie XY Shi LS. Comparison of outcomes between neoadjuvant imatinib and upfront surgery in patients with localized rectal GIST: An inverse probability of treatment weighting analysis. J Surg Oncol (2021) 124(8):1442–50. doi: 10.1002/jso.26664 34494280

[23] Demetri GD van Oosterom AT Garrett CR Blackstein ME Shah MH Verweij J. Efficacy and safety of sunitinib in patients with advanced gastrointestinal stromal tumour after failure of imatinib: a randomised controlled trial. Lancet (London England) (2006) 368(9544):1329–38. doi: 10.1016/S0140-6736(06)69446-4 17046465

[24] Demetri GD Reichardt P Kang YK Blay JY Rutkowski P Gelderblom H. Efficacy and safety of regorafenib for advanced gastrointestinal stromal tumours after failure of imatinib and sunitinib (GRID): an international, multicentre, randomised, placebo-controlled, phase 3 trial. Lancet (London England) 2013, 381(9863):295–302. doi: 10.1016/S0140-6736(12)61857-1 23177515 PMC3819942

[25] Eriksson M Joensuu H. Adjuvant imatinib for GIST: duration likely matters. Ann Oncol (2021) 32(4):434–6. doi: 10.1016/j.annonc.2021.01.073 33524476

[26] Debiec-Rychter M Sciot R Le Cesne A Schlemmer M Hohenberger P van Oosterom AT. KIT mutations and dose selection for imatinib in patients with advanced gastrointestinal stromal tumours. Eur J Cancer (Oxford England:1990) (2006) 42(8):1093–103. doi: 10.1016/j.ejca.2006.01.030 16624552

[27] Dhillon S. Avapritinib: First approval. Drugs (2020) 80(4):433–9. doi: 10.1007/s40265-020-01275-2 32100250

